# Bone marrow inflammatory memory in cardiometabolic disease and inflammatory comorbidities

**DOI:** 10.1093/cvr/cvad003

**Published:** 2023-01-19

**Authors:** Ioannis Mitroulis, George Hajishengallis, Triantafyllos Chavakis

**Affiliations:** Institute for Clinical Chemistry and Laboratory Medicine, Faculty of Medicine, Technische Universität Dresden, 01307 Dresden, Germany; First Department of Internal Medicine and Department of Haematology, Democritus University of Thrace, 68100 Alexandroupolis, Greece; Department of Basic and Translational Sciences, Laboratory of Innate Immunity and Inflammation, Penn Dental Medicine, University of Pennsylvania, Philadelphia, PA 19104, USA; Institute for Clinical Chemistry and Laboratory Medicine, Faculty of Medicine, Technische Universität Dresden, 01307 Dresden, Germany; Centre for Cardiovascular Science, QMRI, University of Edinburgh, Edinburgh EH16 4TJ, UK

**Keywords:** trained immunity, cardiovascular risk, hematopoietic stem and progenitor cells, clonal hematopoiesis

## Abstract

Cardiometabolic disorders are chief causes of morbidity and mortality, with chronic inflammation playing a crucial role in their pathogenesis. The release of differentiated myeloid cells with elevated pro-inflammatory potential, as a result of maladaptively trained myelopoiesis may be a crucial factor for the perpetuation of inflammation. Several cardiovascular risk factors, including sedentary lifestyle, unhealthy diet, hypercholesterolemia, and hyperglycemia, may modulate bone marrow hematopoietic progenitors, causing sustained functional changes that favour chronic metabolic and vascular inflammation. In the present review, we summarize recent studies that support the function of long-term inflammatory memory in progenitors of the bone marrow for the development and progression of cardiometabolic disease and related inflammatory comorbidities, including periodontitis and arthritis. We also discuss how maladaptive myelopoiesis associated with the presence of mutated hematopoietic clones, as present in clonal hematopoiesis, may accelerate atherosclerosis *via* increased inflammation.

**This article is part of the Spotlight Issue on Obesity, Metabolism, and Diabetes.**

## 1. Introduction

Cardiovascular disorders, including ischemic heart disease and stroke, are the leading causes of death worldwide, whereas diabetes mellitus is also included in the top 10 list of death causes globally.^1^ During the COVID-19 pandemic, the presence of diabetes and cardiovascular disease (CVD) was associated with more severe outcome, further underscoring the impact of metabolic and cardiovascular disorders in public health.^2^

Chronic, non-resolving inflammation is integral to the pathogenesis of cardiometabolic disorders and contributes to the progression of atherosclerosis.^3^ Monocytes are a key cellular population in atherogenesis, since these cells migrate into atherosclerotic lesions, in a process that involves integrins such as α4β1, and the C-C chemokine receptor type 2 (CCR2) and CCR5,^4^ where they differentiate to macrophages, which are the most abundant inflammatory cells in the atherosclerotic plaque.^4,5^ In parallel to several studies in animal models that describe the role of chronic inflammation and inflammatory monocytes/macrophages in atherothrombosis,^5^ acute inflammation is also linked to cardiovascular events, such as acute myocardial infarction^3^ and stroke.^6^ Cells of the innate arm of the immune system, including monocytes and neutrophils, are established mediators of this acute inflammatory response by responding to initial danger signals released during ischemia-reperfusion injury at the sites of vascular occlusion.^7–9^

The link between chronic inflammation and cardiovascular morbidity is convincingly established by clinical observations and interventional studies. To this end, patients with chronic inflammatory arthritis, such as rheumatoid and psoriatic arthritis, systemic lupus erythematosus, or the oral mucosal inflammatory disease periodontitis have increased risk for cardiovascular events.^10–12^ Accordingly, enhanced attention to cardiovascular risk management is recommended for such patients.^13^ Additionally, anti-inflammatory treatment of patients with previous myocardial infarction or patients with chronic coronary disease with colchicine^14,15^ or treatment of patients after myocardial infarction that had a C-reactive protein concentration > 2 mg/L with canakinumab,^16^ an antibody against interleukin (IL)-1β, resulted in decreased risk for occurrence of subsequent cardiovascular events. To address the mechanism behind the favourable effect of colchicine administration, targeted proteomic analysis was performed in sera from patients with history of acute coronary syndrome under treatment with colchicine; this approach revealed a reduction in the levels of 37 proteins after 30 days of treatment, including IL-18, IL-1 receptor antagonist, IL-6, and the neutrophil-specific proteins proteinase 3 and myeloperoxidase.^17^ These clinical findings suggest that modulation of inflammation could be a possible effective therapeutic strategy for patients with cardiovascular disease.

Myeloid immune cells are produced by hematopoietic stem and progenitor cells (HSPC) in a process defined as myelopoiesis.^18,19^ HSPCs express receptors for a variety of inflammatory factors, including cytokines, growth factors and pathogen-derived molecular patterns, enabling them to react upon inflammation or infection in peripheral organs with enhanced generation of myeloid cells; the latter mechanism is called emergency myelopoiesis.^18^ This process has a central role in the replenishment of cells of innate immunity during acute systemic infections, thereby ensuring that adequate numbers of granulocytes as well as monocytes are generated for promoting the defense against invading pathogens.^20^ For this reason, several cytokines, such as type I and II interferons, tumour necrosis factor (TNF) and IL-1,^18,20–22^ and myeloid lineage-specific growth factors, including monocyte colony-stimulating factor (M-CSF) and granulocyte-macrophage (GM)-CSF,^18,22–24^ drive directly the proliferative expansion and myeloid lineage differentiation of HSPCs. This effect is mediated through the activation of myeloid lineage-specific transcription factors in HSPCs, such as PU.1 by IL-1 and TNF and STAT1 by type I interferons.^20^ In the case of chronic inflammatory stimulation, hematopoietic stem cells (HSC) re-enter, however, a quiescent state, in order to protect their self-renewal capacity and prevent the depletion of their pool.^25^ Besides the crosstalk between inflammatory mediators and HSPCs in the context of reconstitution of the peripheral immune cell pool under stress conditions, it was recently recognized that inflammatory agonists may also induce long-term alterations in HSPCs, rendering them hyper-responsive to secondary stimuli, thereby resulting in a form of innate immune memory.^26^ Innate immune memory or trained immunity defines that specific triggers may drive enhanced inflammatory preparedness and responsiveness in cells of innate immunity to secondary heterologous challenges; this process may be initiated already at the level of progenitors in the bone marrow, hence termed central trained immunity.^27–29^ The present review focuses on the cardinal function of bone marrow hematopoietic progenitors in the establishment of inflammatory memory and its implication in pathology and progression of cardiometabolic inflammation.

## 2. Rewiring of HSPC in cardiometabolic disorders

**Figure 1:**
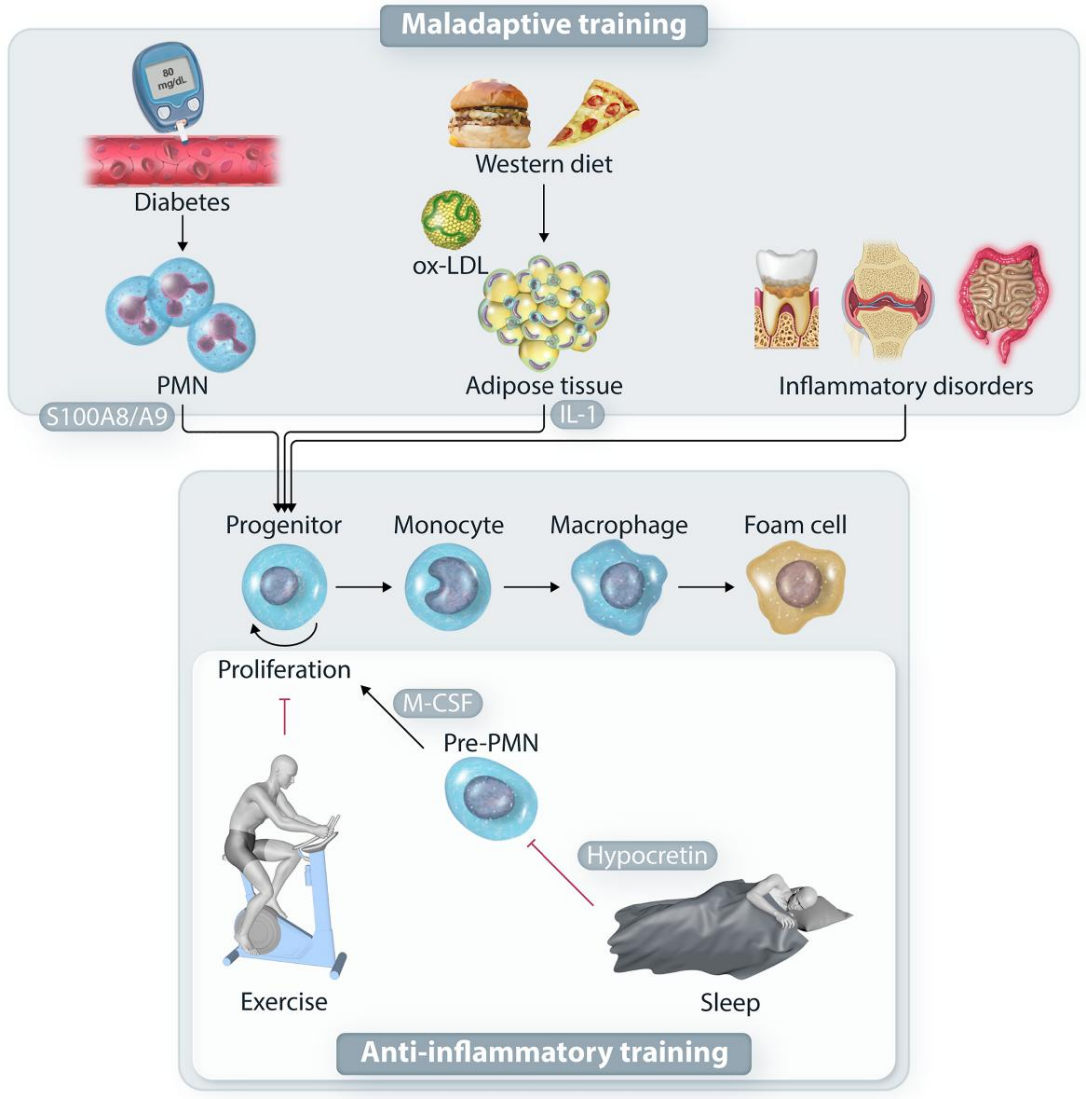
Rewiring of myelopoiesis in atherosclerotic disease. Systemic inflammatory disorders, as well as cardiometabolic risk factors, including diabetes, hyperglycemia and hypercholesterolemia drive maladaptive myelopoiesis, which results in the production of inflammatory monocytes and macrophages that can give rise to foam cells. Activated neutrophils in diabetes or adipose tissue macrophages in obesity and hypercholesterolemia drive the activation of hematopoietic progenitors in the bone marrow. On the other hand, healthy life style exerts an anti-inflammatory effect by blocking hematopoietic progenitor proliferation. In this regard, a healthy sleep, through hypothalamic hypocretin release and suppression of M-CSF production by neutrophil precursors, as well as exercise, suppress the maladaptive myelopoiesis in the context of atherosclerotic disease.

Substantial evidence supports that atherosclerosis, disorders associated with increased atherogenic risk and acute cardiovascular events are linked to enhanced myelopoiesis (*Figure 1*). A seminal recent study by Rohde et al. demonstrated that bone marrow HSCs, defined by flow cytometry as lineage^−^CD34 ^+^ CD38^−^CD45RA^lo^CD90^+^ cells, from patients with atherosclerosis and hypertension or post myocardial infarction show increased proliferation rate compared to controls.^30^ Additionally, increased proliferation rate was observed in common myeloid progenitors (CMP; lineage^−^CD34 ^+^ CD38^int^CD45RA^−^CD123^int^) and granulocyte-macrophage progenitors (GMP; lineage^−^CD34 ^+^ CD38^int^CD45RA ^+^ CD123^int^) from patients with hypertension alone, hypertension and atherosclerosis or post myocardial infarction compared to control.^30^ These findings suggest that the enhanced myelopoiesis, which characterizes cardiovascular disorders and may fuel inflammation, is instigated at the level of HSCs.

Several mouse models have been engaged in an effort to identify the mechanisms that drive myelopoiesis by atherogenic risk factors and in cardiometabolic disorders. One of the multiple mechanisms, by which defects in cholesterol metabolism drive atherogenesis, may involve HSPC functional alterations. Studies in mice susceptible to the development of diet-induced atherosclerosis proposed a direct role for cholesterol metabolism in regulating the proliferative potential of HSPCs.^31^ Specifically, inhibition of efflux of cholesterol in hematopoietic progenitors owing to the absence of ATP-binding cassette transporters ABCG1 and ABCA1 results in increased proliferative expansion of HSPCs and hence elevated myelopoiesis.^32^ This was associated with altered membrane properties and an upregulation of the cell surface expression of the common β subunit of the IL3/GM-CSF-receptor.^32^ Moreover, the interaction between apolipoprotein E and these cholesterol efflux systems in HSPCs regulates their proliferation potential, whereas exogenous high-density lipoprotein (HDL) administration suppresses HSPC cycling.^33^ In addition to increased HSPC proliferation in the bone marrow, *Abca1*^−/−^*Abcg1*^−/−^ double deficient mice and *Apoe*^−/−^ mice show enhanced HSPC mobilization and extramedullary myelopoiesis, which may further contribute to the increased generation of inflammatory myeloid cells.^34^ These findings suggest that cholesterol levels regulate extramedullary myelopoiesis, which is a significant source of inflammatory monocytes that infiltrate the atherosclerotic plaques further promoting atherosclerosis.^35^ Along the same line, Gu et al. further showed that low-density lipoprotein (LDL) levels were correlated with the frequency of mobilized CD34^+^ HSPCs in the peripheral blood of healthy subjects and that the protein expression of SREBP2, the master transcriptional regulator of cholesterol biosynthesis, was increased in HSPCs from patients with hypercholesterolemia.^36^ HSPC mobilization has been also shown in *Apoe*^−/−^ mice after coronary artery ligation, which thereby resulted in extramedullary myelopoiesis and monocyte production in the spleen.^37^ Sympathetic nervous system activity was implicated in this process.^37^ Interestingly, via a similar mechanism, social stress may drive HSPC mobilization and splenic myelopoiesis through β-adrenergic signaling.^38^

Increased cholesterol levels and decreased expression of *Abca1*, *Abcg1*, and *Apoe* were observed in HSPCs from mice subjected to collagen-induced arthritis and the K/BxN serum-transfer model of inflammatory arthritis^39^; importantly the presence of rheumatoid arthritis correlates with increased cardiovascular risk.^13^ Mice subjected to arthritis models display enhanced bone marrow and extramedullary myelopoiesis; additionally, treatment of isolated progenitor cells with a cocktail of inflammatory factors that are implicated in rheumatoid arthritis disease pathogenesis, including GM-CSF, TNF, IL-6, and IL-1β, resulted in a downregulation of the expression of the aforementioned cholesterol efflux-associated genes, thereby suggesting an intimate crosstalk between the inflammatory milieu of the disease and cholesterol metabolism in HSPCs.^39^ Consistently, administration to mice of β-glucan, a fungal-derived inflammatory stimulus that drives trained immunity, was also shown to decrease the expression of *Abca1* and increase the expression of *Ldlr* and of genes involved in cholesterol biosynthesis in HSPCs, which was also accompanied by higher cell proliferation and myeloid lineage bias of HSPCs.^22^ Diabetes mellitus and associated hyperglycemia, a major cardiometabolic risk factor, has been also associated with HSPC activation and enhanced myelopoiesis^26^ (*Figure 1*). Increased monocyte numbers, due to enhanced proliferation of myeloid progenitor cells, have been described in two mouse models of type I diabetes mellitus (T1D) and pancreatic insufficiency, due to pancreatic islet cell destruction.^40^ In these models, the release of the endogenous danger signal proteins S100A8/A9 by neutrophils drives myelopoiesis, through increased release of GM-CSF and M-CSF in the bone marrow.^40^ To address whether transient hyperglycemia, which is common in diabetic patients, despite optimal glycemic control,^41^ can also promote myelopoiesis, Flynn et al. engaged atherosclerotic prone *Apoe*^−/−^ mice and performed bolus glucose administration.^42^ This study demonstrated that intermittent hyperglycemia resulted in increased myelopoiesis and accelerated progression of atherosclerosis.^42^ Interestingly, repeated intraperitoneal glucose administration to mice resulted in increased numbers of myeloid lineage progenitors in the bone marrow after one day, which led to increased monocyte and neutrophil numbers in peripheral blood a week thereafter.^42^ Specifically, glucose uptake by neutrophils, resulted in increased glycolysis and production of the S100A8/A9 alarmins, which in turn promoted myelopoiesis.^42^ This observation suggests that frequent episodes of transient hyperglycemia could drive constant generation of increased numbers of inflammatory cells. Except from the activation of the myelopoiesis progenitor pool, diabetes mellitus has been shown to disrupt the HSPC niche in the bone marrow, altering the HSPC mobilization potential.^43^

Diabetes is associated with poor HSPC mobilization in patients undergoing autologous peripheral blood stem cell transplantation, whereas G-CSF-induced mobilization is impaired in mouse models of diabetes; this phenomenon was attributed to altered expression of CXCL12 and stem cell factor (SCF), which are critical for HSC maintenance within the bone marrow.^44^ Another study linked the enhanced myelopoiesis observed in diabetes mellitus to defective G-CSF-induced mobilization.^45^ This study demonstrated that monocytes and neutrophils in the bone marrow environment of diabetic mice release oncostatin M, which drives CXCL12 production by mesenchymal cells of the HSC niche, resulting in decreased mobilization.^45^ Modulation of HSC niche cells, and specifically enhanced angiogenesis and endothelial cell dysfunction, has been also observed in mouse models of hypertension induced by angiotensin (Ang II), diet-induced atherosclerosis in *Apoe*^−/−^ mice, and myocardial infarction induced by coronary artery ligation.^30^ In the latter study, IL-6 and versican release by bone marrow endothelial cells drove enhanced myelopoiesis in atherosclerosis and myocardial infarction.^30^ Along the same line, chronic sympathetic nervous system-mediated hypertension in mice mediates myelopoiesis by disrupting the bone marrow microenvironment through cleavage of CXCR4 by neutrophil-derived serine proteases.^46^

Obesity, a main driver of type 2 diabetes, is an important cardiometabolic risk factor that links inflammation and myelopoiesis.^47,48^ In obesity-related inflammation, monocytes are recruited to the adipose tissue and differentiate to macrophages with an inflammatory and metabolically-activated phenotype.^47–50^ Several studies have shown that obesity in humans is associated with increased monocyte numbers in peripheral blood,^51,52^ suggesting that monopoiesis is involved in disease pathogenesis. Increased numbers of circulating monocytes and neutrophils have been also observed in leptin-deficient *Ob/Ob* mice and in high fat diet-induced obesity, reflecting enhanced myelopoiesis in the bone marrow.^53^ This activation of the myeloid progenitor compartment was not, however, associated with leptin signalling, since leptin replenishment did not affect monopoiesis.^53^ Additionally, blood glucose reduction using a sodium glucose co-transporter 2 inhibitor did not reduce monocytosis, suggesting that myelopoiesis in obesity is not the result of hyperglycemia,^53^ an observation that is strikingly different to that observed in T1D mouse models.^40^ This study further demonstrated, using fat pad transplant experiments, that inflammatory signalling in the adipose tissue was responsible for the enhanced myelopoiesis.^53^ With a series of bone marrow transfer experiments, using donor cells from *Tlr4*^−/−^, *Myd88*^−/−^, *Nlrp3*^−/−^, and *Il-1r*^−/−^, the authors demonstrated that TLR4/MyD88-dependent activation in the macrophages of the adipose tissue induces the production of IL-1β, which in turn exerts actions within the bone marrow by activating hematopoietic progenitor differentiation into myeloid lineage.^53^ Another study further demonstrated that diet-induced obesity in mice results in an impaired response of HSPCs to chemotherapy-induced hematopoietic stress, associated with decreased quiescence of HSC.^54^ This functional impairment was abolished in *Tlr4*^−/−^ progenitors, revealing a critical role for TLR4 in obesity-associated hematopoiesis.^54^

Exercise in mice may also increase HSC quiescence, which results in decreased myelopoiesis^55^ (*Figure 1*). In *Apoe*^−/−^ mice fed with a western diet, exercise decreased myeloid cell generation and atherosclerosis progression. The effect of exercise on circulating leukocytes was also shown in this study by analysing the clinical data in two cohorts of patients with existing cardiovascular risk.^55^ Disrupted sleep due to obstructive sleep apnea is an additional cardiovascular risk factor.^56^ Even though the exact mechanism that mediates this effect is not known, there is evidence that maladaptive myelopoiesis may play a role in this process.^57^ Specifically, sleep fragmentation in *Apoe*^−/−^ mice accelerates atherosclerosis and enhances myelopoiesis.^57^ Sleep has been shown to suppress myelopoiesis *via* hypothalamic release of hypocretin, as assessed by using a parabiotic mouse model engaging WT mice partnered with mice deficient for hypothalamic hypocretin (*Hcrt*^−/−^), which had suppressed myelopoiesis compared to *Hcrt^−/−^* partnered *Hcrt*^−/−^ mice. *Hcrt^−/−^Apoe^−/−^* mice under high fat diet also display aggravated atherosclerosis. Release of M-CSF by neutrophil precursor cells was further demonstrated to mediate the effect of hypocretin.^57^ In summary, these findings indicate that normalization of myelopoiesis could underlie the beneficial effect of lifestyle modifications on cardiovascular risk.

In addition to metabolic disorders, inflammatory disorders linked to increased cardiometabolic risk have been associated with bone marrow inflammation and dysregulated myelopoiesis (*Figure 1*). Animal studies have clearly demonstrated that enhanced myelopoiesis and activation of HSPCs is a characteristic of experimental arthritis.^58,59^ Similarly, myeloid skewing of HSPCs has been observed in patients and mice with systemic lupus erythematosus (SLE).^60^ Finally, bone marrow inflammation has been found in patients with periodontitis, a disorder that is also associated with cardiometabolic risk.^61^

## 3. Trained immunity, cardiovascular, and comorbid disorders

**Figure 2:**
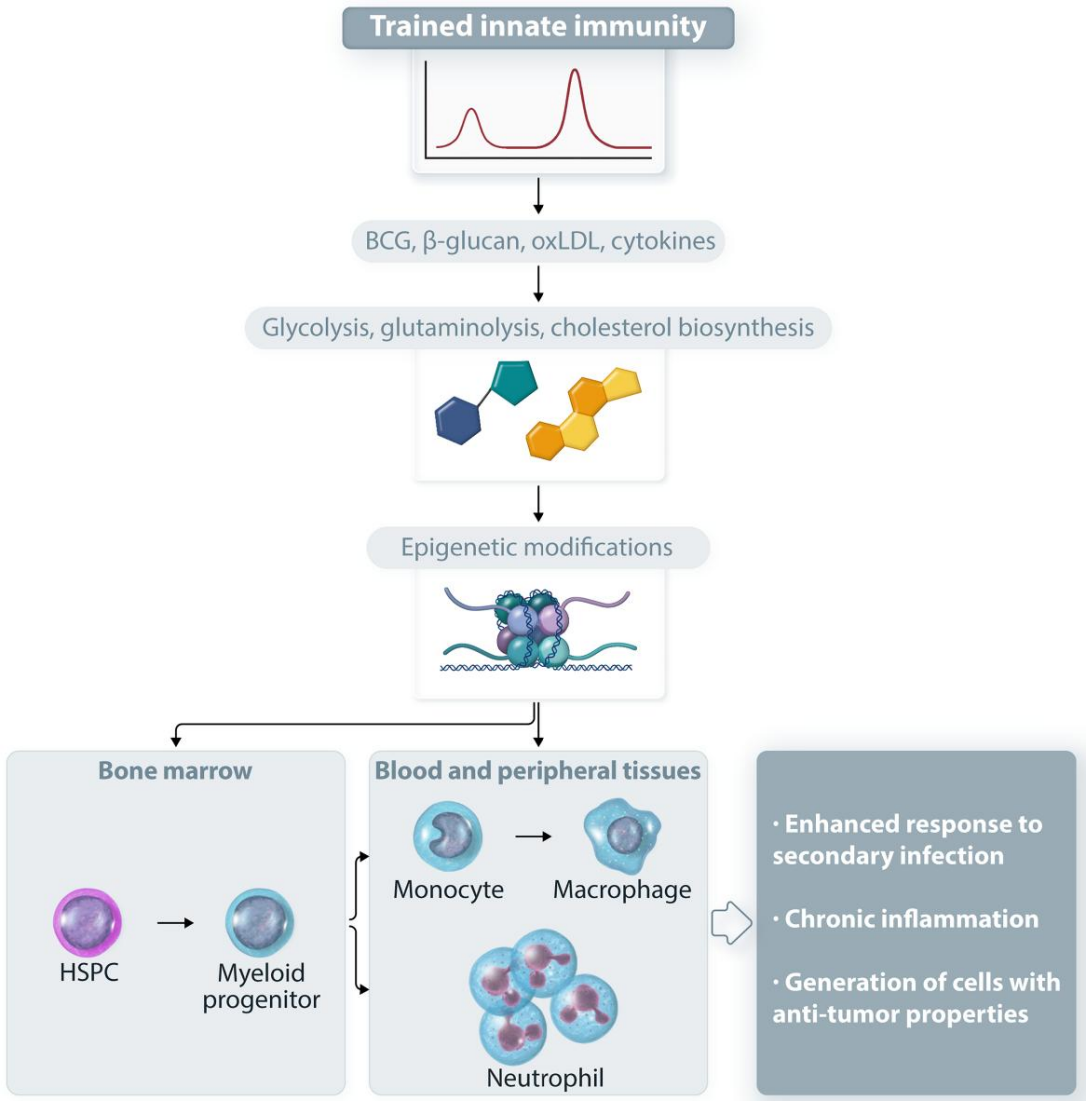
Central and peripheral trained innate immunity. Stimuli, including the BCG vaccine, fungal β-glucan, oxLDL or cytokines, such as IL-1, drive metabolic changes that further promote epigenetic modifications in hematopoietic and myeloid progenitors in the bone marrow (central trained immunity) and in mature innate immune cells, including neutrophils and macrophages (peripheral trained immunity). These changes promote the enhanced adaptive response of myelopoiesis to secondary stimuli, which hence results in increased production of trained neutrophils or monocytes that bear enhanced inflammatory preparedness. The induction of trained innate immunity has a protective role against invading pathogens and could be exploited as cancer immunotherapy but may also fuel chronic inflammatory disorders.

As alluded to above, trained immunity is defined as the enhanced response of innate immune cell populations to secondary triggers after an initial activation by the same or an unrelated stimulus.^27^ Certain infectious agents or vaccines have been initially shown to functionally reprogram innate immune cells, e.g. monocytes or natural killer cells, to strongly respond to secondary infections, conferring better protection of the host.^27^ Substantial evidence suggests that alterations in cell metabolism and epigenetic rewiring act in concert for triggering trained immunity, since a series of enzymes that mediate epigenetic reprogramming are activated or inhibited by metabolic intermediates^27,29^ (*Figure 2*). Trained immunity engages signalling pathways related to the mechanistic target of rapamycin (mTOR) and hypoxia-inducible factor 1α (HIF1a), switching metabolism from oxidative phosphorylation to glycolysis.^62^ Glutaminolysis further provides substrates for the tricarboxylic acid (TCA) cycle, which results in accumulation of TCA intermediates, such as fumarate, that can induce epigenetic changes in monocytes.^63^ Interestingly, activation of cholesterol biosynthesis pathway and generation of mevalonate has been also implicated in trained immunity.^64^

Besides differentiated immune cells, inflammatory modulation of HSPCs contributes to central trained immunity^26^ (*Figure 2*). As HSPCs are long-lived cells, central trained immunity may explain the sustained trained immunity effects thought to last for at least a few months. Preclinical studies in animal models have shown that prototypic trained immunity inducers, such as bacillus Calmette–Guérin (BCG) vaccine or β-glucan can drive long-term functional changes in HSPCs, instructing them towards myeloid lineage differentiation.^22,65^ The functional modulation of HSPCs by trained immunity-associated stimuli was further shown to have a beneficial effect on the HSPC response to chemotherapy-induced hematopoietic stress^22^ or to secondary mycobacterial infections.^65,66^ Evidence from mouse models and human studies showed that the epigenetic modifications that occur in progenitor cells upon trained immunity induction can be further detected in the progeny, giving rise to monocytes/macrophages^66,67^ or granulocytes^68,69^ with trained properties. For instance, the neutrophil phenotype resulting from β-glucan-induced trained granulopoiesis has anti-tumour activity.^68^ Several inflammatory mediators have been detected as mediators of the actions of trained immunity on HSPCs, including IL-1,^66^ type I^68^ or type II interferons,^65^ and GM-CSF.^22^

Except from the prototypic stimuli that trigger trained immunity, stimuli associated with cardiometabolic disorders may also train mature myeloid cells, myeloid progenitors, and HSPCs.^70,71^ Oxidized low-density lipoprotein (oxLDL), a critical pro-atherogenic molecule, is known to induce long-term changes in monocytes, which thereby acquire an inflammatory phenotype and form foam cells.^72^ In a similar manner, western type diet in *Ldlr^−/−^* mice induced long-lasting functional modulation of GMPs, rendering them hyper-responsive to secondary *in vivo* challenge with lipopolysaccharide.^73^ This functional reprogramming of GMPs was associated with their epigenetic and transcriptional rewiring, making the functional modulation of myelopoiesis persistent even after switch to normal diet.^73^ This modulation of GMPs in response to western diet was abolished in *Nlrp3^−/−^Ldlr^−/−^* mice, showing that NLRP3 inflammasome and IL-1β play a critical role in this process.^73^ This study further demonstrated that *Nlrp3* deficiency resulted in decreased atherosclerosis,^73^ suggesting that inflammasome and IL-1β are critical players in atherogenesis. Whether IL-1β acts through trained myelopoiesis or engages other cell types and/or mechanisms has to be interrogated in future studies. Despite the experimental evidence that oxLDL drives myelopoiesis, blocking of cholesterol biosynthesis by statins in patients with familial hypercholesterolemia did not alter monocyte hyper-responsiveness to a secondary TLR activation, despite the reduction of LDL levels.^74^ Hyperglycemia has been also shown to induce long-lasting functional changes in macrophages and their progenitors in the bone marrow.^75^ This study further demonstrated that the functional alterations induced by hyperglycemia can be traced back to HSC by generating bone marrow chimeras using donor cells from normoglycemic mice or streptozotocin-induced diabetic mice and transferring them into *Ldlr*^−/−^ recipient mice fed a western diet. Recipient mice that received transplantation of hematopoietic progenitors from diabetic mice showed more severe atherosclerotic plaque formation, suggesting that hyperglycemia drives long-lasting modifications in donor HSCs, which are responsible for the enhanced vascular inflammation in recipients post-transplantation.^75^

Based on the aforementioned studies, there is substantial evidence that cardiometabolic risk factors, such as hypercholesterolemia and diabetes can induce trained immunity-associated changes in HSPCs, which in turn fuel inflammation and worsen the atherosclerotic process. On the contrary, exercise can promote HSPC fitness.^55^ Exercise in mice has been demonstrated to promote HSPC repopulation potential and lineage output, which lasted for several weeks.^55^ This form of HSPC memory was linked to epigenetic changes; HSPCs from exercising mice displayed decreased chromatin accessibility, which particularly affected genes that regulate HSPC expansion and lineage commitment.^55^ Interestingly, in addition to its effect on atherogenesis, exercise has a beneficial effect on emergency myelopoiesis in a mouse model of lipopolysaccharide-induced systemic inflammation; exercise also resulted in increased myelopoiesis and improved survival in mice subjected to a sepsis model induced by cecal ligation and puncture.^55^ This latter observation suggests that exercise drives a beneficial type of memory in hematopoiesis.

## 4. Maladaptively trained myelopoiesis links inflammatory comorbidities

Recent evidence suggests that bone marrow-based trained immunity can causally link inflammatory comorbidities and explain their reciprocal association.^76^ This concept was established in the context of periodontitis and arthritis, two inflammatory diseases associated with elevated risk of CVD. However, the underlying mechanisms go beyond and above periodontitis and arthritis and are likely relevant to multiple inflammatory diseases driven by myeloid cells.

Periodontitis is an inflammation-related disease of the tooth-supporting tissues that is linked to elevated risk of CVD and other systemic inflammatory pathologies, such as type 2 diabetes (T2D), non-alcoholic fatty liver disease (NAFLD), and rheumatoid arthritis (RA).^12,61,77,78^ Although periodontitis has common genetic and acquired (*e.g*. aging, smoking, adiposity, and diabetes) risk factors with CVD and other chronic inflammatory disorders, the independent association of periodontitis with its inflammatory comorbidities continues to be present even after adjusting for those confounders.^79^ This is because the local dysbiosis and immune response of the host within the periodontal tissue are drivers of systemic low-grade inflammation that may in turn influence the development of comorbid diseases. Systemic inflammation related to periodontitis (*e.g*. elevated IL-1, IL-6, C-reactive protein, and blood neutrophil counts) is likely caused by the spillover of locally produced inflammatory cytokines into the circulation and by hematogenous translocation of periodontal pathogens that can pass through the ulcerated epithelium into the bloodstream causing clinically documented bacteremias.^12,80^ Periodontitis-related bacteremias can be frequent as they are caused not only during professional dental care (*e.g*. probing or mechanical debridement) but also during daily activities, such as chewing, toothbrushing, and flossing.^12,80^

Failure to exercise oral hygiene to control the tooth-associated microbial biofilm was associated with increased systemic inflammation and risk for CVD in a prospective study.^81^ On the other hand, successful treatment of periodontitis causes reduction of systemic inflammatory markers.^12,82–84^ Periodontal treatment also improves vascular and kidney functions,^83,85^ reduces HbA1c and glucose plasma levels in T2D patients,^83^ and decreases LDL and triglyceride levels in hyperlipidemic patients with periodontitis.^86^ Moreover, long-term improvement of periodontal health was associated with mitigated progression of intima-medial thickness of the carotid artery.^87^ The connection between periodontitis and CVD (and other comorbidities) is bidirectional. Several studies suggest that the prevalence of periodontitis increases in the presence of systemic comorbidities (*e.g*. CVD, and T2D).^79,88^ Consistently, systemic inflammation (using as markers white blood cell counts and fibrinogen levels) was associated longitudinally with the severity of periodontal disease.^89^

Using positron emission tomography/computed tomography with 2-deoxy-2-[fluorine-18]fluoro-D-glucose (^18^F-FDG-PET/CT), which detects sites of inflammatory activity,^90^ several studies correlated periodontal disease with arterial inflammation^91–93^ that was confirmed histologically by assessing macrophage infiltration in dissected carotid artery plaques.^93^ Moreover, inflammation in the periodontium, as revealed by ^18^F-FDG uptake, was associated with enhanced risk of ensuing cardiovascular events.^92^ Intriguingly, the use of ^18^F-FDG-PET/CT also revealed a correlation between inflammation of the gingiva and hematopoietic activity in the bone marrow, indicative of stimulated myelopoiesis.^91^ Indeed, ^18^F-FDG-PET/CT-determined bone marrow hematopoietic activity was correlated with increased white blood cell and monocyte counts (albeit not with erythrocytes).^91^ These findings suggest the operation of a periodontitis-bone marrow inflammatory axis that correlates with comorbid inflammation, although causation and directionality are uncertain.

**Figure 3:**
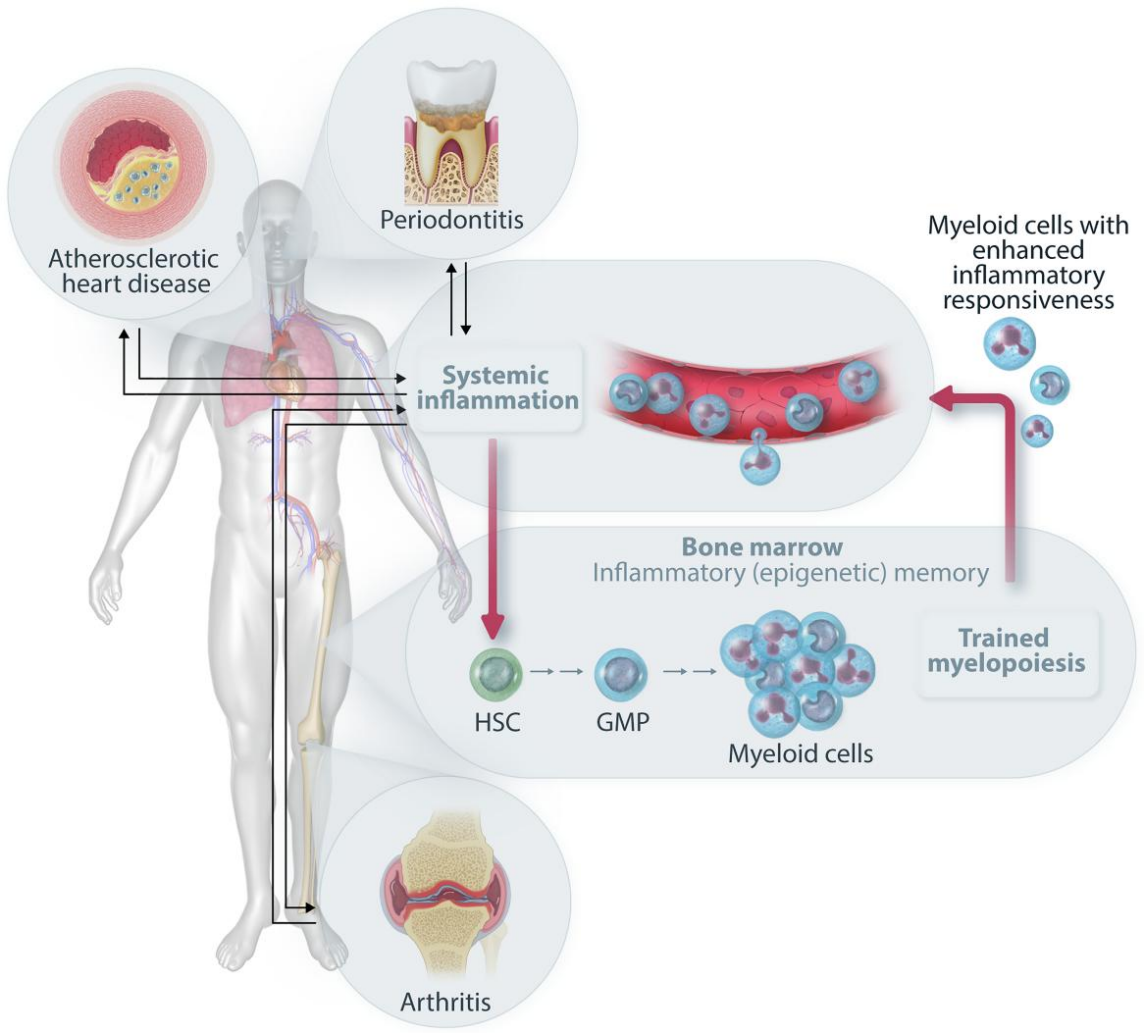
Trained immunity in the bone marrow as a common mechanism for inflammatory comorbidities. Systemic inflammation (*e.g*. due to an inflammatory disease) may induce epigenetically based inflammatory memory in HSPCs, which thereby preferentially undergo myeloid-skewed differentiation giving rise to increased numbers of hyper-responsive myeloid cells (trained myelopoiesis). These trained neutrophils or monocytes/macrophages can infiltrate to sites of infection and/or inflammation (*e.g*. periodontium, atherosclerotic plaques, and joints) and may contribute to the initiation or progression of inflammatory diseases that emerge as comorbidities. In this regard, patients with periodontitis have increased risk of developing arthritis and atherosclerosis. The trained myeloid cells also promote the chronicity of these conditions by setting off a feed-forward loop of reciprocally reinforced bidirectional interactions between the peripheral inflamed tissues and the bone marrow.

We have previously suggested that periodontitis-related systemic inflammation may induce trained immunity of the bone marrow HSPCs, promoting the enhanced generation of myeloid cells with hyper-responsive properties that may not only exacerbate periodontitis but also promote inflammatory pathology of comorbidities, such as CVD.^18,61^ In other words, systemic inflammation-induced epigenetic reprogramming of HSPCs towards trained myelopoiesis might perpetuate inflammation and generate a vicious cycle linking bone marrow and comorbid inflammatory disorders.^18,61^ We have recently demonstrated this concept experimentally, that is, that maladaptive training of myelopoiesis underlies inflammatory comorbidities (*Figure 3*). We exemplified this novel principle in the setting of the periodontitis-RA axis.^76^

Specifically, experimental periodontitis-induced systemic inflammation in mice led to epigenetic rewiring of HSPCs, which were imprinted with a myeloid-differentiation bias. Upon future challenge, the trained HSPCs gave rise to higher numbers of monocytes and neutrophils with enhanced inflammatory preparedness. This periodontitis-trained phenotype could be transmitted by transplantation of trained HSPCs to naïve recipients, which displayed increased joint inflammation and pathology when subjected to collagen antibody-induced arthritis (CAIA), as compared to CAIA-subjected controls transplanted with untrained HSPCs.^76^ The reverse was also demonstrated, *i.e*. CAIA similarly induced maladaptive training of HSPC and led to enhanced severity of periodontitis in bone marrow transplanted mice. The periodontitis-induced maladaptive training of myelopoiesis was crucially dependent on IL-1 signalling in HSPCs; indeed, training failed in mice with HSPC-specific deletion of IL-1 receptor.^76^ This finding suggests that systemic inhibition of IL-1 or the IL-1 receptor could suppress the maladaptive training of HSPCs and thereby block a central causal mechanism for inflammation chronicity and inflammatory comorbidities. It is intriguing to speculate that the beneficial results of IL-1β neutralization in the treatment of atherosclerosis in the CANTOS trial^16^ could, in part, be attributed to blockade of trained myelopoiesis in the bone marrow, in other words, atherosclerosis might also be exacerbated by a maladaptively trained bone marrow.

Periodontitis-related innate immune training of the bone marrow has not been formally demonstrated in humans so far. However, besides the clinical imaging studies suggesting the existence of a periodontitis-bone marrow inflammatory axis (discussed above), additional studies are consistent with this concept. Myeloid cells in the peripheral blood from patients suffering from periodontitis respond with higher production of pro-inflammatory cytokines to *ex vivo* re-stimulation with lipopolysaccharide or whole bacteria than cells from periodontally healthy individuals, even if the patients have undergone successful periodontal therapy.^94,95^ In general, multiple studies indicate that individuals with periodontitis have higher numbers of peripheral blood neutrophils that display altered and hyper-reactive functions upon *ex vivo* challenge, such as reduced anti-oxidant responses and increased release of reactive oxygen species and granular enzymes.^94,96–101^ Furthermore, peripheral blood mononuclear cells from patients suffering from coronary artery disease display higher production of cytokines upon *ex vivo* stimulation, as compared to cells deriving from healthy individuals; additionally, transcriptome analysis of these patients’ hematopoietic progenitors showed enrichment for neutrophil- and monocyte-related pathways suggestive of a myeloid-differentiation bias.^102^ These data imply generation of innate immune memory and are reminiscent of findings from BCG vaccinated individuals whose circulating monocytes displayed long-term hyper-responsiveness in terms of cytokine release following *ex vivo* stimulation with different bacterial or fungal organisms.^67,103^ This enhanced hyper-responsiveness was, at least partially, attributed to BCG-imprinted epigenetic adaptations in HSPCs, thereby leading to increased myelopoiesis.^67^

Similar to the clinical imaging studies discussed above in the context of periodontitis-CVD comorbidity, a subpopulation of RA patients being in clinical remission shows increased bone marrow hematopoietic activity and arterial wall inflammation (both assessed by a study engaging ^18^F-FDG uptake and PET/CT).^104^ These findings indicate that RA remission does not automatically reduce the CVD risk of these patients and further imply a common mechanism for RA and CVD based on maladaptive innate immune training occurring in the bone marrow. Inflammatory memory may therefore perpetuate sustained myelopoiesis in RA patients despite being in remission, thereby maintaining their higher risk for CVD. Conversely, CVD can also enhance myelopoiesis^47,71^ and potentially lead to generation of bone marrow inflammatory memory, thereby exacerbating RA.

Except for the direct effect on myeloid cell populations or their precursors, trained immunity can also indirectly modulate the adaptive immune cell functions; conversely, T cells may also regulate the induction of trained innate immunity. For instance, CD8^+^ T cells support the induction of memory in alveolar macrophages upon viral infection *via* IFN-γ production.^105^ This local induction of trained immunity in the lungs enabled improved response to secondary bacterial pneumonia through enhanced neutrophil chemotaxis into the lungs.^105^ To the opposite direction, the enhanced production of pro-inflammatory cytokines, such as IL-1β, TNF of IL-6, as a result of induction of trained immunity can shape T cell function, regulating T cell differentiation and polarization, and as a result, Th1/Th2 and Th17/Treg balance.^106^ This interplay between trained cells of the myeloid lineage with T cells could be an additional link between inflammatory disorders and CVD comorbidities, since adaptive immune cells play a critical role both in autoimmune diseases such as RA^107^ and metabolic diseases, such as T2D and obesity.^108,109^

In conclusion, induction of a maladaptive form of trained immunity in bone marrow HSPCs represents a potentially unifying mechanism underlying development of multiple comorbidities, including the elevated risk of several systemic inflammatory diseases in periodontitis patients.

## 5. Ageing, clonal hematopoiesis, inflammation, and cardiovascular risk

Ageing is associated with immune dysregulation and inflammation, which in turn is associated with increased incidence of inflammatory and metabolic disorders.^110^ One of the several effects of age on the innate immune system is the increase and the myeloid bias and differentiation of hematopoietic progenitors both in mice and humans,^111–113^ resembling, in parts, the changes that take place in the bone marrow upon maladaptive myelopoiesis associated with inflammatory and metabolic disorders. Even though BCG-induced trained immunity results in downregulation of inflammation in elderly people one month after injection,^114^ a potential effect of trained immunity on the functional decline of HSC in the elderly population has not been studied. Yet, this interesting observation could be exploited by further studies, in order to address whether induction of trained immunity by BCG administration could dampen inflammation associated with disorders that have increased prevalence in the elderly population. Interestingly, it has been reported that ageing is associated with loss of epigenetic regulation in HSC,^112^ which could prevent the proper induction of epigenetic modifications that take place during induction of trained immunity.

Ageing is also linked to increased risk for clonal hematopoiesis. Specifically, the use of next generation sequencing in hematology enabled the identification of otherwise healthy subjects with hematopoietic clones derived from HSCs. The prevalence of this condition increases with age, affecting more than 10% of individuals with an age higher than 70 years and approximately 20% of those older than 90 years.^115^ Given the high prevalence of this condition in the elderly population in the absence of evident hematologic disease or cytopenia, the term clonal hematopoiesis of indeterminate potential (CHIP) or age-related clonal hematopoiesis (ARCH) has been introduced.^116,117^ The identified clones in CHIP are hallmarked by the existence of mutations in genes that are related to myeloid malignancies, including *TET2*, *DNMT3A, ASXL1*, and *JAK2,* with a variant allele frequency (VAF) ≥ 2%.^116^

CHIP was initially associated with increased all-cause mortality, which, however, could not be attributed to increased progression towards myeloid malignancies.^115^ A follow-up study revealed a clear association between CHIP and coronary artery disease as well as early-onset myocardial infarction.^118^ Analysis of samples from two prospective cohorts revealed that the risk of coronary artery disease was almost two-times increased in individuals with CHIP compared to the control group.^118^ Additionally, this study provided a clear link between clonal hematopoiesis and myocardial infarction, by using samples derived from two previous studies and showing that the risk was four-times higher in subjects with CHIP.^118^ Since then, several studies have linked CHIP with cardiovascular disorders.^117^ For instance, the identification of *ASXL1*, *TET2*, and *JAK2* in CHIP has been associated with heart failure irrespectively of previously documented coronary artery disease.^119^ The association between CHIP and mortality in patients with ischemic heart failure has also been assessed.^120^ This study reported that the five-year-mortality increased from 18% in patients without mutation in *DNMT3A* or *TET2* to 42% in patients with mutations in both genes.^120^ Additionally, increased clonal size, as defined by the VAF, was also associated with increased mortality.^120^ Interestingly, patients with COVID-19 and CHIP had a two-time higher risk for severe outcome,^121^ providing a plausible explanation for the worse outcome of COVID-19 in the elderly population and in patients with CVD.^2^ Another study evaluated the prevalence of CHIP and its association with cardiovascular events in patients with SLE.^122^ This study demonstrated that CHIP occurred in patients with SLE 20 years earlier than in the control group, without having any independent association with cardiovascular events, which, however, could be due to the limited number of patients.^122^

In addition to genetic risk factors,^123^ CHIP has also been associated with modifiable cardiovascular risk factors. A study in samples from 8709 postmenopausal women from the Women’s Health Initiative studied the link between CHIP and lifestyle factors linked with cardiovascular risk, including body mass index, smoking, physical activity, and diet quality. In this study, unhealthy lifestyle, obesity, and smoking were associated with CHIP.^124^ Another large study in a cohort of 44 111 individuals from the UK biobank further addressed whether diet quality could be associated with the development of CHIP.^125^ This study revealed that the prevalence of CHIP was significantly higher in individuals with unhealthy diet (7.1%), characterized by consumption of red meat, processed food, and high salt intake above median, compared to those with healthy diet habits (5.1%).^125^ A possible explanation for this observation could be low-grade inflammation caused by western diet that can affect hematopoietic progenitors and cause long-term functional changes in myeloid cells.^73^

The mechanism that links CHIP with cardiovascular events has been extensively studied. The initial study that reported for the first time the association between CHIP and CVD further demonstrated that transplantation of bone marrow cells from *Tet2*^−/−^ mice into *Ldlr^−/−^* mice resulted in enhanced atherosclerosis, kidney glomerulosclerosis, and inflammatory infiltrates in the liver,^118^ suggesting that *Tet2*-deficient clones accelerate the inflammatory process related to cardiometabolic disease. Using a similar approach, Fuster et al. performed partial bone marrow reconstitution of bone marrow in *Ldlr^−/−^* mice with cells from *Tet2*^−/−^ mice to create a mouse model of CHIP that mimics the coexistence of normal and mutated clones. This study reported that the increased formation of atherosclerotic plaques in recipient mice that received *Tet2*-deficient bone marrow cells could be attributed to increased IL-1β production by *Tet2*-deficient macrophages.^126^ Along the same line, *Tet2*-deficiency in mice accelerated heart failure in two mouse models of heart failure.^127^ In this study, myocardial remodelling and fibrosis, which result in ischemic heart failure, were more prominent in mice that underwent partial transplantation with bone marrow cells from *Tet2*^−/−^ mice in a model of infarction of the myocardium and a model of cardiac pressure overload.^127^ Pharmacological inflammasome inhibition partially reversed this phenotype, suggesting the involvement of IL-1β in this process.^127^ Another study used as donor cells bone marrow lineage-negative cells with CRISPR-mediated inactivation of *Tet2* and *Dnmt3a* genes and demonstrated that the inactivation of both genes promotes Ang II-induced cardiac hypertrophy and fibrosis.^128^ Furthermore, CRISPR-mediated mutations in *Ppm1d* gene, which are related to clonal hematopoiesis in patients previously treated with chemotherapeutic drugs for cancer, had a similar effect on cardiac remodelling after Ang II administration.^129^ In addition to heart failure, partial transplantation with *Tet2*-deficient cells worsened insulin resistance in aged and obese mice.^130^ Increased levels of IL-1β were observed in the white adipose tissue of these mice, whereas pharmacological inhibition of NLRP3 inflammasome ameliorated insulin resistance in these mice.^130^ Chimeric *Ldlr*^−/−^ mice bearing mutated *Jak2^V617F^* also demonstrated accelerated atherosclerosis.^131^ This effect was, however, inhibited when bone marrow cells from *Jak2^V617F^ Aim2*^−/−^ double mutant mice were used, suggesting that AIM2 inflammasome and IL-1β play a critical role in this process.^131^ Therapeutic intervention with Anakinra, an IL-1 receptor antagonist, was further shown to decrease the necrotic core of atherosclerotic lesions, suggesting that IL-1β inhibition is a therapeutic target at least in this preclinical model.^131^

In addition to these preclinical studies, clinical observations suggest that inflammation, and especially IL-1β, are a major link between CHIP and increased cardiovascular risk. For instance, it has been reported that CHIP carriers have significantly higher levels of C-reactive protein compared to non-carriers, even in the subgroup of patients with known CVD.^132^ Using samples from the CANTOS trial that evaluated the effect of canakinumab in patients with atherosclerotic disease after myocardial infarction and high levels of C-reactive protein,^16^ it was shown that IL-1β neutralization with canakinumab may be more beneficial for patients with CHIP associated with *TET2* mutations, compared to non-carriers of CHIP-associated mutations.^133^

According to these preclinical and clinical observations, mutated hematopoietic clones are associated with a higher inflammatory potential that drives cardiometabolic disease. Conversely, recent findings have revealed that atherosclerosis-associated inflammation drives clonal hematopoiesis.^134^ Engaging mathematical models, Heyde et al. demonstrated that the increased proliferation rate in HSC in patients with atherosclerosis could result in a 3.5 fold risk for CHIP by the age of 70.^134^ In the mouse chimeric model of CHIP, *Tet2*-deficient clones had a growth advance in *Ldlr*^−/−^ recipient mice under atherogenic diet or after sleep deprivation.^134^ This experimental evidence indicates that there is a vicious cycle linking CHIP and inflammation; this could provide an explanation for the earlier occurrence of CHIP in SLE patients, a disease that is common in females at the childbearing age, when CHIP is extremely rare.^122^

## 6. Conclusion

Cardiometabolic diseases are the primary cause of mortality worldwide; hence, it is imperative to better characterize the molecular mechanisms that drive atherogenesis. Several modifiable cardiometabolic risk factors, including hyperlipidemia, hyperglycemia, or even unhealthy nutrition, as well as inflammation associated with several comorbidities have been shown to modulate HSPC function, resulting in maladaptive myelopoiesis that promotes inflammation and subsequent increased atherogenesis. Additionally, the increased cardiometabolic risk in individuals with CHIP further supports the central role of HSPC adaptations in atherogenesis. Contrastingly, the beneficial effects of exercise may be mediated by improved HSPC fitness due to exercise. Taken together, the bone marrow is an important hub orchestrating systemic inflammatory responses that are involved in atherogenesis and CVD. The generation of epigenetic inflammatory memory in the bone marrow could provide an at least partial causal explanation underlying the association of CVD with inflammatory comorbidities.
